# Automated assessment of balance: A neural network approach based on large-scale balance function data

**DOI:** 10.3389/fpubh.2022.882811

**Published:** 2022-09-21

**Authors:** Jingsong Wu, Yang Li, Lianhua Yin, Youze He, Tiecheng Wu, Chendong Ruan, Xidian Li, Jianhuang Wu, Jing Tao

**Affiliations:** ^1^College of Rehabilitation Medicine, Fujian University of Traditional Chinese Medicine, Fuzhou, China; ^2^Fujian Collaborative Innovation Center for Rehabilitation Technology, Fuzhou, China; ^3^Shenzhen Institutes of Advanced Technology, Chinese Academy of Science, Shenzhen, China; ^4^University of Chinese Academy of Sciences, Beijing, China

**Keywords:** neural networks, machine learning, feature selection, balance, automated assessment

## Abstract

Balance impairment (BI) is an important cause of falls in the elderly. However, the existing balance estimation system needs to measure a large number of items to obtain the balance score and balance level, which is less efficient and redundant. In this context, we aim at building a model to automatically predict the balance ability, so that the early screening of large-scale physical examination data can be carried out quickly and accurately. We collected and sorted out 17,541 samples, each with 61-dimensional features and two labels. Moreover, using this data a lightweight artificial neural network model was trained to accurately predict the balance score and balance level. On the premise of ensuring high prediction accuracy, we reduced the input feature dimension of the model from 61 to 13 dimensions through the recursive feature elimination (RFE) algorithm, which makes the evaluation process more streamlined with fewer measurement items. The proposed balance prediction method was evaluated on the test set, in which the determination coefficient (R2) of balance score reaches 92.2%. In the classification task of balance level, the metrics of accuracy, area under the curve (AUC), and F1 score reached 90.5, 97.0, and 90.6%, respectively. Compared with other competitive machine learning models, our method performed best in predicting balance capabilities, which is especially suitable for large-scale physical examination.

## Introduction

Balance ability refers to the ability to maintain a stable posture immediately and autonomously when a person's center of gravity deviates (1, 2). The problem of falls has become a global public health issue because it greatly increases the risk of injury and even death among middle-aged and elderly people (1–6). Studies have found that balance impairment (BI) is a key factor in causing people to fall (7, 8). With the aging of the international society, it is particularly important to conduct early assessment and screening of the balance ability of middle-aged and elderly patients.

The research of balance ability has always been the focus of some scholars (9–16). Many studies have achieved satisfactory results by using machine learning or deep learning methods to solve the problem of balance prediction and falling (see Supplementary Table 3). Yeh et al. (9) developed a virtual reality (VR) balance rehabilitation training system for patients and used the support vector machine (SVM) algorithm to train a machine learning model on data collected from 48 patients and 36 normal people. Khandoker et al. (10) proved the effectiveness of wavelet-based and multi-scale analysis in the assessment of balance disorders in the elderly. Begg et al. (11) collected the minimum foot clearance (MFC) data from 30 young people and 28 old people and analyzed the difference in a dynamic balance between young and elderly. Moreover, as early as 1993, Holzreiter and Köhle (16) used neural networks to analyze healthy and pathological gaits to explain balance assessment.

In addition to the gait perspective, Liu and Cheng (13) started from the center of gravity and used the SVM algorithm to train a fall detection model. Their accuracy rate is as high as 98.4%. Similarly, Bao et al. (14) trained the 1–5 PT (physical therapist) level evaluation model and compared it with the actual PT level of physical therapy, and their classification accuracy reached 82%. Different from the above method, Nait et al. (15) used wearable sensors to obtain daily data of 296 elderly people and combined the convolutional neural network with long-term and short-term memory to build a network model, which can effectively assess the risk of falls based on wearable sensor data. Pickle et al. (17) used a dynamic non–linear autoregressive neural network to train motion data collected from five able-bodied individuals and five individuals with Parkinson's disease walking on a non-steady-state locomotor circuit comprising stairs, ramps, and changes of direction. They found that estimating segment contributions to angular momentum from mechanical signals (linear acceleration, angular velocity) from a sparse set of body segments is a feasible method for assessing coordination of balance. Dubois et al. (18) used the Microsoft Kinect sensor to collect the balance of 84 participants in 8 balanced tasks. Using the clustering algorithm to analyze the experimental data, they found that the prediction results of standing on the foam pad are the most accurate.

A large amount of literature and reviews on gait and balance ability (9–18) has discussed the role and importance of gait and balance ability in falls (19), but few papers study how to optimize balance test items and quickly measure balance function. At present, balance test system is the commonly used tool for quickly measuring and evaluating the balance ability of subjects, such as German Bismarck Super Balance (GBSB) (20, 21) and Biodex Balance System (22). The GBSB system uses three-dimensional force measurement to eliminate the interference factor of the individual's weight on the force plate, which can obtain more accurate measurement data. There are four actions measured by the GBSB system, namely (1) stand on feet with eyes open (FEO); (2) stand on feet with eyes closed (FEC); (3) stand on one foot with eyes open (OFEO); (4) stand on one foot with eyes closed (OFEC). Each set of actions includes 12 test items as shown in Supplementary Table 2. In addition, each action should be kept for a while to collect enough test data. For each subject, it takes at least 90 sec to measure the four sets of actions. At the same time, taking into account the understanding and execution efficiency of instructions for the elderly, when they performing the balance test, the data collection time will be extended, which brings inconvenience to large-scale physical examinations and BI screening. In addition, in order to obtain a comprehensive balance function result, balance assessment requires the measurement of a large number of metrics. Specifically, GBSB system needs to measure at least 61 metrics of the subject to calculate the balance score (20, 21). However, there is massive redundancy in these metrics.

In recent years, artificial intelligence has been applied in various fields of medical data processing (23, 24). Using machine learning algorithms to mine potential information in data has become one of the promising tools for solving medical problems (25–31). In this work, we aim at building a balance assessment model based on the data-driven machine learning techniques. After analyzing the collected data set, we will propose an artificial neural network (ANN) model based on machine learning to predict balance capabilities. Finally, the proposed model is able to quickly and automatically evaluate new subjects, which can simplify the measurement metrics and ensure high accuracy.

## Methodology

### Overview

The overall process of balance prediction is shown in Supplementary Figure 1. Subjects need to stand in different positions on the GBSB platform for data collection, such as bipedal and single-foot tests with eyes open or closed. Then the system will automatically measure the four sets of items. After that, we clean and preprocess the collected data to facilitate subsequent training and prediction using neural networks. We used BS and BL as the ground truth for regression and classification tasks. A lightweight artificial neural network model was proposed to accurately predict the balance ability of new test examples.

### Data collection and preprocessing

The dataset was obtained from the Second Affiliated Hospital of Fujian University of Traditional Chinese Medicine, China. This dataset was used for retrospective analysis, and a total of 17,541 subjects aged 12–80 years entered the analysis with medical examination data. This dataset does not show any personal privacy other than medical information. The study was approved by the Medical Ethics Committee of the Second Affiliated Hospital of Fujian University of Traditional Chinese Medicine, China (No. SPHFJP-K2019059-02).

The features consist of 48 common measurement features of four specific actions and 13 other features. The equipment used for data collection is the GBSB system. The subjects were required to stand on the instrument in different postures to do four sets of actions according to the instructions, including FEO, FEC, OFEO, and OFEC. The durations were 30, 30, 20, and 10 sec, respectively. This protocol is set according to the GBSB system, through which we obtain various measurement data of the subjects. The details of test were shown in Supplementary Figure 2.

This dataset collected a total of 17,541 subjects, each with 61-dimensional features and two labels, where the 61 features represent measurement items, and two labels collectively represent the balance ability: Balance Score (0–100) and Balance Level (high, medium, low), which are used as ground truth for regression tasks and classification tasks, respectively. The Balance Score is measured by GBSB system, and then the doctor evaluates the Balance Level according to the Balance Score and the physical condition of the subjects.

As shown in Supplementary Figure 3, the whole 61-dimensional features include 48 common measurement features of four sets of actions and 13 other individual features. Each set of actions includes 12 test items. Supplementary Table 2 shows the value range of 48 common measurement features and four sets of actions.

Eighty percentage of the data is used as the training set, 10% of the data is used as the validation set, the remaining 10% are test set. We performed a 10-fold cross-validation. Since there are missing values in the dataset, which will affect the prediction performance of the model on the balance ability. Firstly, we calculated the median of all samples to deal with the missing values. Because the proportion of missing data is relatively small (account 5% of all), the median is a good way to fit the original data distribution. Regarding the problem of inconsistent feature dimensions, since the original data conforms to the Gaussian distribution, we have carried out the following standardization processing, so that the mean of the data is 0 and the variance is 1.


(1) $$\begin{array}{l} z=\frac{x-\mu }{\sigma }. \end{array}$$

Where *x* is the original value, *z* is the standardized value, μ means the average of all samples, and σ represents the standard deviation of *x*.

### The proposed ANN model

Due to the high accuracy and strong robustness of the neural network algorithm, we designed a model based on a deep neural network to predict the balance ability. To make the neural network model fit the data better, generally, the common methods prefer to increase the depth and width of the network. However, this will increase the number of network parameters exponentially, making the computational complexity of the model greater. Through a large number of experiments, we find that when the number of hidden layers of the neural network is set to five, the accuracy and complexity of the model can be effectively balanced. Supplementary Figure 4 shows the proposed neural network structure. The entire network contains an input layer, five hidden layers, and an output layer. In each middle-hidden layer, the number of neurons is set as 128, 64, 32, 16, and 8 respectively. The dimension of the input layer matches the number of features of the data, while the output layer represents the prediction result of the neural network. We implemented regression and classification tasks using the proposed model. In the classification task, the output layer dimension is the number of categories, which indicates the high, medium, or low level of predicted balance ability. While in the regression task, the output layer has only one dimension, namely the balance score.

Notably, the three important components of a neural network are weights, bias term, and activation function. It is those weight parameters that are constantly updated during the learning process of the network that make the neurons between adjacent layers fully connected. The strength of the connection between neurons is determined by the value of weight. Furthermore, bias is a crucial parameter of the model to ensure that the output value calculated by the input cannot be activated casually. Typically, the activation function acts as a non-linear mapping, which can limit the output amplitude of the neuron within a certain range. The structure of one neuron is shown in the dotted box in Supplementary Figure 4. Given the input feature data *x*_*i*_(*i* = 0, 1, …*n*), the output S is calculated as follows:


(2) $$\begin{array}{l} S=\max\left\{0,\left(w_{0}x_{0}+w_{1}x_{1}+\ldots +w_{n}x_{n}+\text{b}\right)\right\}, \end{array}$$

where the weight parameter *w*_*i*_(*i* = 0, 1, …*n*) represents the connection strength between neurons. It should be noted that the activation function is set in our experiment as the ReLU function, which not only promotes the non-linear representation of features but also makes the neurons have sparseness that further improves the fitting ability of the model.

The purpose of training the model is to obtain the model parameters that minimize the cost function. The cost function (15) we define is as follows:


(3) $$\begin{array}{l} \text{J}\left(\text{w},\text{b}\right)=\frac{1}{\text{m}}\sum_{i=1}^{m}\text{L}\left({ŷ}_{i},y_{i}\right)+\frac{\lambda }{2\text{m}}||\text{W}{||}_{2}^{2}. \end{array}$$

Where m is the total number of samples, ŷ_*i*_ and *y*_*i*_ represent the predicted value and true value of the *i*-th sample respectively and L means cross-entropy loss function. The $\frac{\lambda }{2\text{m}}||\text{W}{||}_{2}^{2}$ is an L2-regularization term used to penalize complex models. And the item λ is a hyperparameter that controls the degree of penalty. In the process of model parameter updating, we use the Adam optimizer to perform gradient descent and back-propagation.

## Experiments

### Experimental settings and metrics

The experimental environment is a Windows 10 system with python 3.6. The neural network model framework used in the experiment is based on scikit-learn library with version 0.24, an efficient tool for data mining which built on numpy, scipy, and matplotlib. In the process of training the neural network, the batchsize is set to 200. The optimizer used for weight update is Adam, and its constant learning rate is initialized to 0.001.

We use common evaluation metrics to evaluate classification and regression models, namely: Accuracy (Acc), Recall (Re), Precision (Pr), F1-score (F1), and coefficient of determination (*R*^2^) score. These evaluation metrics range from 0 to 1. The larger the value, the better the model effect. These formulas are as follows:


(4) $$\begin{array}{l} \text{Acc}=\frac{\text{TP}+\text{TN}}{\text{TP}+\text{TN}+\text{FN}+\text{F}{\text{P}}^{\text{′}}} \end{array}$$

(5) $$\begin{array}{l} \mathrm{Pr}=\frac{\text{TP}}{\text{TP}+\text{F}{\text{P}}^{\text{′}}} \end{array}$$

(6) $$\begin{array}{l} \text{Re}=\frac{\text{TP}}{\text{TP}+\text{F}{\text{N}}^{\text{′}}} \end{array}$$

(7) $$\begin{array}{l} \text{F}1=\frac{\text{TP}\left(2\text{TP}+\text{FP}+\text{FN}\right)}{2\left(\text{TP}+\text{FN}\right)\left(\text{TP}+\text{FP}\right)}. \end{array}$$

(8) $$\begin{array}{l} {\text{R}}^{2}\left(\text{y},\hat{\text{y}}\right)=1-\frac{\sum_{\text{i}=1}^{\text{n}}{\left({\text{y}}_{\text{i}}-{\hat{\text{y}}}_{\text{i}}\right)}^{2}}{\sum_{\text{i}=1}^{\text{n}}{\left({\text{y}}_{\text{i}}-\bar{\text{y}}\right)}^{2}}. \end{array}$$

Where $\bar{y}=\frac{1}{n}\sum_{i=1}^{n}y_{i}$. The ${\hat{y}}_{i}$ and *y*_*i*_ represent the predicted value and true value of the *i*-th sample. TP, TN, FP, and FN represent true positive, true negative, false positive, and false negative, respectively.

### Feature selection

**Figure 1:**
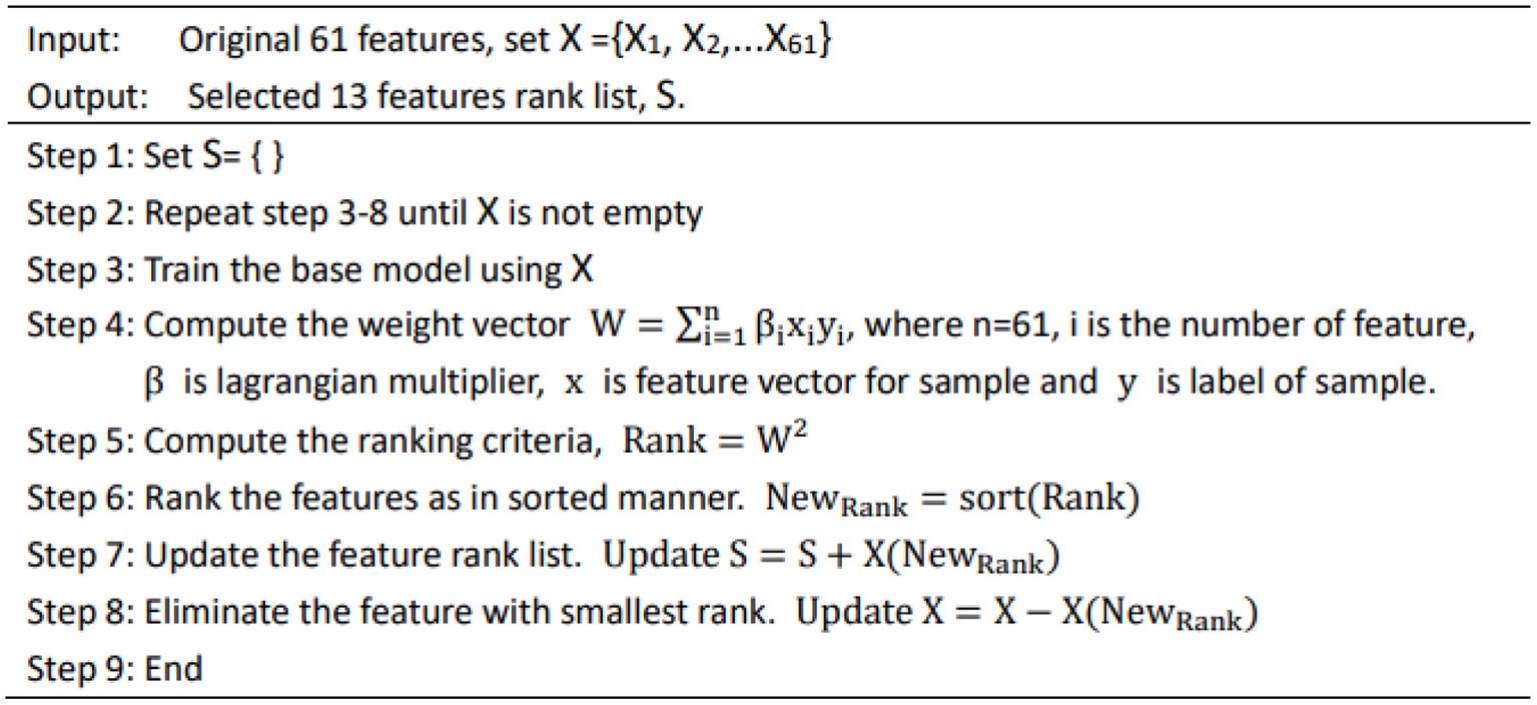
Feature selection algorithm.

Since the data contains irrelevant feature attributes, it will interfere with the prediction of new data. In order to reduce the input feature dimension of the model and improve the robustness of the algorithm, we adopted a recursive feature elimination (RFE) algorithm to filter the 61-dimensional features. As shown in Figure 1, the flow of feature selection algorithm included recursive feature elimination, neural network training, and classification.

## Results

In this study, we used a physical examination database from the clinic, which contains the physical examination data of 17,541 subjects. The descriptive statistics about the subjects is shown in Supplementary Table 1. Among all subjects, the middle-aged and elderly (aged between 41 and 80) accounted for 68.17%. Further, among the people with low and medium balance ability, the middle-aged and elderly (aged between 41 and 80) accounted for 78.36%, indicating that the target population of our proposed method is more suitable for the middle-aged and elderly.

**Figure 2:**
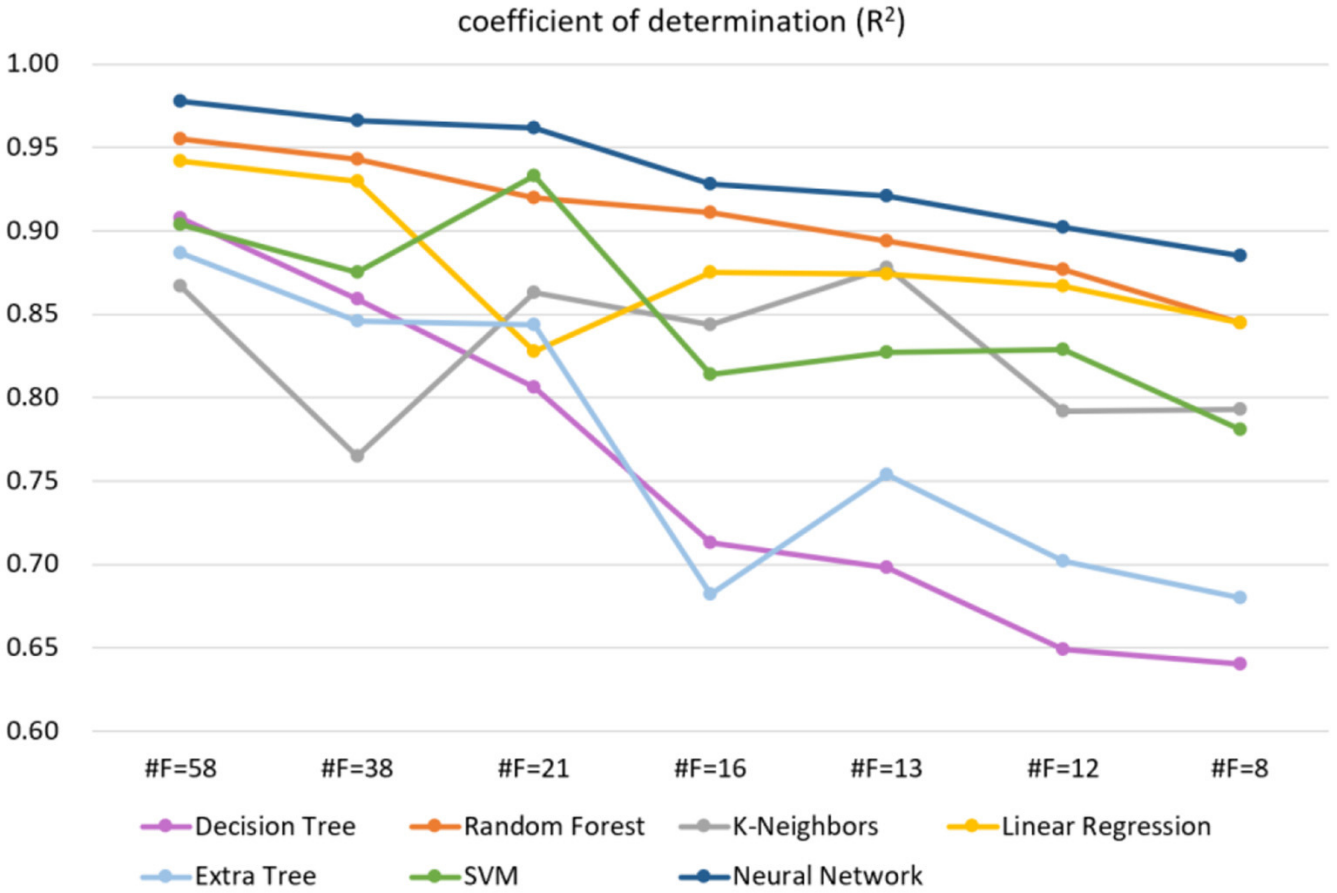
The trend of different methods in the case of reduced feature dimensions, where #F indicates the number of feature dimensions. The higher the R^2^, the better the model effect. The label used on the regression task is balance score (0–100).

A total of 61 balance indicators were measured and entered into analysis. Based on the features selected by the RFE algorithm, we compared the accuracy of different machine learning models on the test dataset. As shown in Supplementary Table 4, these competitive machine learning algorithms include Decision Tree, Random Forest, K-Neighbors, Linear Regression, Extra Tree, and Support Vector Machine. Compared with other methods, our proposed neural network model performs best regardless of the input feature dimension. Typically, when the feature is 61-dimensional, the coefficient of determination of our model is the highest, reaching 97.8%, while the feature is reduced to 13 dimensions, our model is still the best, with the coefficient of determination reaching 92.2%. Figure 2 further shows the trend of different methods in the case of reduced feature dimensions. It can be seen from Figure 2 that our neural network model can still maintain a high coefficient of determination even when the feature dimension drops sharply, indicating that our method is more robust and stable.

By weighing the feature dimension and model accuracy, that is to make the measurement time of subject as low as possible while keeping the coefficient of determination as high as possible, we finally chose #F = 13 as the final filtered feature. The selected 13-dimensional features are shown in Table 1. Taking the selected 13 features as the input of the proposed neural network model, the true and predicted balance score of samples on the test set are shown in Supplementary Figure 5. In order to show more intuitively, we only drew 100 samples. We can see from the resulting graph that the predicted balance score is basically the same as the true value, which shows the accuracy of the model from an intuitive effect.

**Table 1:** The selected 13 features.

**#**	**Features**	**Units**	**Description**	**Range**	**Mean**
1	TLS FEC	mm	Total trajectory length of shaking, eyes closed with feet	43.1–2,065.2	266.3
2	TLS OFEC	mm	Total trajectory length of shaking, eyes closed with one foot	26.6–3,799.1	506.2
3	PA OFEC	mm^2^	Peripheral area, eyes closed with on one foot	5.2–57,102.7	2469.2
4	TLPA FEC	-	Track length per unit area, eyes closed with feet	0.1–27.2	2.0
5	TLPA OFEC	-	Track length per unit area, eyes closed with on one foot	0.0–6.2	0.3
6	Y-D FEC	mm	Y-axis mean center displacement, eyes closed with feet	−68.5–84.1	27.7
7	Y-D OFEC	mm	Y-axis mean center displacement, eyes closed with one foot	−120.4–127.1	13.9
8	AS-X FEC	mm/s	Average speed in the X-direction, eyes closed with feet	0.6–65.9	32.1
9	AS-X OFEC	mm/s	Average speed in the X-direction, eyes closed with one foot	0.7–259.3	182.6
10	AS-Y FEC	mm/s	Average speed in the Y-direction, eyes closed with feet	1.2–60.0	30.2
11	AS-Y OFEC	mm/s	Average speed in the Y-direction, eyes closed with one foot	2.1–240.8	48.9
12	LT-X OFEC	mm	Length of track in the X-direction, eyes closed with one foot	7.1–2,593.1	1,008.7
13	LT-Y FEC	mm	Length of track in the Y-direction, eyes closed with feet	36.7–1,800.8	947.1

As shown in Table 2, compared with other machine learning models like Decision Tree (32), Linear Discriminant Analysis (LDA) (34), K-Neighbors (36), Logistic (38), Naive Bayes (40), and Support Vector Machines (SVM) (42), our method has the highest accuracy, recall, precision, and F1-score evaluation metrics, reaching 90.5, 90.8, 90.5, and 90.6%, respectively.

**Table 2:** Classification results (%) with evaluation metrics of different methods on 13-dimensional features.

**Single method**	**Acc**	**Pr**	**Re**	**F1**	**Ensemble method**	**Acc**	**Pr**	**Re**	**F1**
Decision tree (32)	78.4	78.4	78.4	78.4	BDT (33)	84.7	85.0	84.7	84.8
LDA (34)	78.6	78.9	78.6	78.4	RF (35)	84.0	84.4	84.0	84.1
K-neighbors (36)	80.8	81.3	80.8	80.9	ET (37)	78.9	72.7	72.7	72.7
Logistic (38)	80.9	80.9	80.9	80.8	SGBoost (39)	87.3	87.5	87.3	87.3
Naive bayes (40)	60.1	79.0	60.1	53.4	AdaBoost (41)	83.1	83.6	83.1	83.2
SVM (42)	83.5	83.9	83.5	83.6	Voting (43)	86.7	86.9	86.7	86.7
Ours	90.5	90.8	90.5	90.6	Ours	90.5	90.8	90.5	90.6

The label used on the classification task is the balance level (high, medium, low). Acc, accuracy; Pr, precision; Re, recall; F1, F1-score.

From the perspective of clinical balance assessment procedures, compared with traditional measurement methods, the proposed method has the following advantages, namely (1) fewer measurement metrics, (2) less measurement time. Our method only needs to measure 13 metrics to evaluate the balance ability, which takes only 40 sec (55% less time than GBSB's 90 sec). For the evaluation accuracy, the determination coefficient *R*^2^ of our method reached 92.2%, and the classification accuracy rate reached 90.5%, both of which met the clinical requirements.

## Discussions

In this work, we propose the method to assess the balance ability efficiently and accurately, which can predict the risk of falling. To obtain the balance score and balance level, we implemented regression and classification tasks on the data set respectively by designing and training a neural network model. Generally, the fitting ability of neural networks increases as the number of layers deepens, but the training time and complexity of the model also increase. We weighed these two aspects and designed a neural network with five hidden layers. To prevent the model from overfitting, we added an L2 regular term to the cost function to prevent the weight parameter from being too large, which is conducive to improving the generalization ability and robustness of the neural network.

It is known that the data and features determine the upper limit of machine learning, while models and algorithms only approach the upper limit. Therefore, feature selection helps to discover the output results that we are interested in. We filtered the 61-dimensional features based on the RFE algorithm. As shown in Table 2, the accuracy of different models on the test set decreases as the feature dimensions decrease. Specifically, when the feature dimension was reduced from 61 to 38, which was reduced nearly by half, but the accuracy of the model remained almost unchanged, indicating that the original data contained feature attributes that were not related to balance ability. It is worth noting that when the feature dimension of our model is reduced from 61 to 13, the accuracy rate drops from 97.8 to 92.2%. The feature dimension is reduced by 78.7%, however, the model accuracy is only reduced by 5.7%. The above situation illustrates our proposed neural network can maintain high accuracy even when the feature dimension is drastically reduced, suggesting that the original data is redundant and our model is robust.

**Figure 3:**
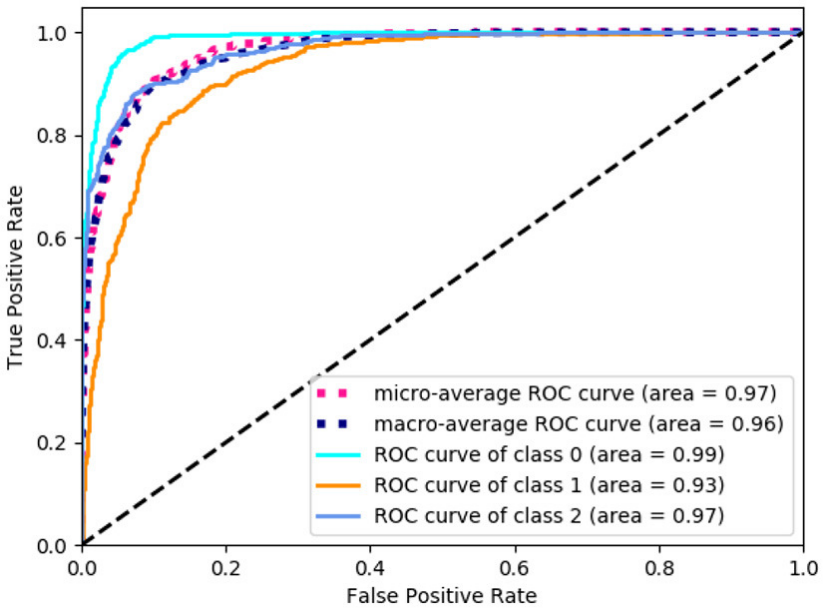
The area under the curve (AUC) value of each balance level. The class 0, 1, and 2 correspond to low, medium and high balance levels respectively.

In order to get the level of balance ability, a comprehensive study of different methods on classification task has been undertaken. There are three categories for the level of balance ability in the data set, namely: high, medium, and low. For the prediction of the balance level, we changed the output layer dimension of the proposed neural network to 3 to perform the classification task. The input to the network is the selected 13-dimensional features in Table 1. As shown in Table 2, compared with other machine learning models like Decision Tree (32), Linear Discriminant Analysis (LDA) (34), K-Neighbors (36), Logistic [ (38)], Naive Bayes (40), and Support Vector Machines (SVM) (42), our method has the highest accuracy, recall, precision, and F1-score evaluation metrics, reaching 90.5, 90.8, 90.5, and 90.6%, respectively. For the balance level, we count the area under the curve (AUC) value of each category, as shown in Figure 3. Class 0, 1, and 2 correspond to low, medium, and high balance levels respectively, and their AUC values reach 99, 93, and 97%, which shows the neural network model trained by 13-dimensional features has high accuracy and low redundancy.

In machine learning, every single algorithm has different characteristics and application range. To absorb the advantages of different models, integration technology is usually used to combine multiple algorithms for improving the accuracy of the model. There are three methods of integration: bagging, boosting, and voting.

### Bagging

**Figure 4:**
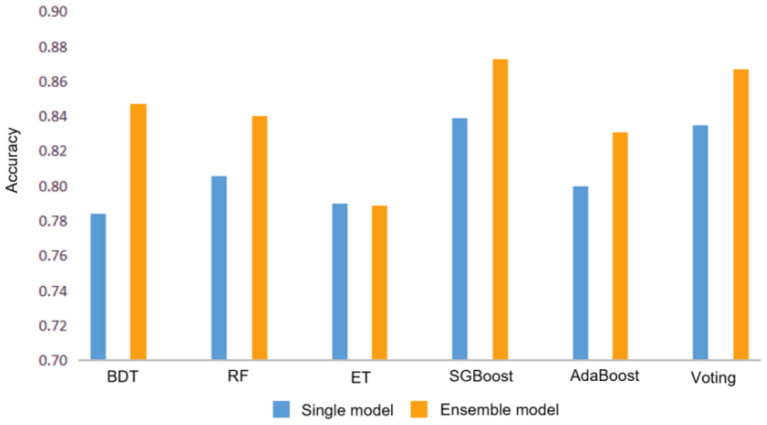
Accuracy comparison between single model and ensemble model. Where BDT, RF, and ET represent Bagged Decision Tree, Random Forest and Extra Tree respectively.

The bagging algorithm separates the training data set into multiple subsets by random sampling with a return. Then each subset trains a weak model. Finally, the weight of each weak model is averaged by combining strategies to obtain a strong model. The models using the bagging method include Bagged Decision Tree (BDT) (33), Random Forest (RF) (35), and Extra Tree (ET) (37). As shown in Table 2, their accuracy reaches 84.7, 84.0, and 78.9% respectively. As can be seen from Figure 4, except for Extra Tree, there are significant improvements in the bagging models.

### Boosting

The boosting algorithm first trains the data set according to the initialized weight *D*1 to obtain a weak model and then the weak model updates the weight *D*1 according to the error to obtain the weight *D*2. In the second round, *D*2 is used to train the data, and so on. The main idea of boosting is to train multiple models and form a sequence. Each model in the sequence corrects the errors of the previous one and finally merges all weak models to obtain a strong model. The models that use boosting include AdaBoost (41) and SGBoost (39), and their accuracy reaches 83.1 and 87.3%, respectively, which is better than the overall effect of the bagging algorithm.

### Voting

The voting algorithm (43) creates two or more models, uses voting to package the algorithm, and then calculates the average prediction of each sub-model. The voting algorithm shown in Table 2 is a strong classifier obtained by voting on three single models including a decision tree classifier, a support vector machine, and a logistic regression model.

Compared with ensemble techniques, the accuracy of our method is about 3% higher than the second-place SGBoost method, which proves the neural network model we proposed can obtain the best performance whether dealing with regression tasks or classification tasks. The main reason for this is that our neural network model can approximate any non–linear function, skip the model analysis and directly mine the relationship among the data. In addition, the neural network fully considers the influence of characteristic factors, while other machine learning models are relatively fixed. Moreover, the neural network allows outliers in the training data, which has strong robustness and fault tolerance to noise. The above discussion illustrates the fact that our method can effectively extract different dimensions of features to better fit and predict the balance ability.

### Limitations

Although the proposed method shows good performance, there are still some aspects that can be further explored in the future. The first is limited adaptability. Since the data collected is local balance data, the prediction model may not apply to other regions. In this case, prediction models in other regions require additional training on local data sets to generate new models. Secondly, there is room for improvement in the accuracy of our automated assessment because of the noise in raw data. We can also optimize the model through grid search and random search to further improve accuracy.

## Conclusions

In this work, we proposed an artificial neural network model to train large-scale physical examination data, so that the model can efficiently and accurately predict the balance score and balance level, which is beneficial for the early screening and prevention of falls in the middle-aged and elderly. Compared with other competitive machine learning models, our method performed best in predicting balance capabilities, where the determination coefficient of balance score reaches 92.2%. In the classification task of balance level, the metrics of accuracy, precision, recall, and F1 score reached 90.5, 90.8, 90.5, and 90.6% respectively. The proposed method greatly reduces the dimensionality of the input features, indicating that for the prediction of balance ability, only two actions with 13 items need to be measured to get the result, which greatly reduces the workload.

## Data availability statement

The raw data supporting the conclusions of this article will be made available from the corresponding author upon reasonable request.

## Ethics statement

The studies involving human participants were reviewed and approved by Medical Ethics Committee of The Second Affiliated Hospital of Fujian University of Traditional Chinese Medicine. Written informed consent from the participants' legal guardian/next of kin was not required to participate in this study in accordance with the national legislation and the institutional requirements.

## Author contributions

JiaW and JT secured the fundings and conceived the original idea and designed the study. JinW and LY prepared the ethical reviews application. JinW, LY, YH, TW, CR, and XL were responsible for the data collection and collation. YL did the data modeling and analysis. JinW wrote the manuscript. All authors contributed to further development of this manuscript and approved the final manuscript.

## Funding

This work was supported in part by the Shenzhen Basic Research Program (No. JCYJ20180507182441903), the Key Research and Development Project funded by the Ministry of Science and Technology of the People's Republic of China (Nos. 2019YFC1710301 and 2019YFC1710305), and the Open Research Fund of Fujian Key Laboratory of Rehabilitation Technology (No. KF2019001).

## Conflict of interest

The authors declare that the research was conducted in the absence of any commercial or financial relationships that could be construed as a potential conflict of interest.

## Publisher's note

All claims expressed in this article are solely those of the authors and do not necessarily represent those of their affiliated organizations, or those of the publisher, the editors and the reviewers. Any product that may be evaluated in this article, or claim that may be made by its manufacturer, is not guaranteed or endorsed by the publisher.
