# Supplementary material for: Automated assessment of balance: A neural network approach based on large-scale balance function data
Source: Front Public Health. 2022 Sep 21;10:882811. doi: 10.3389/fpubh.2022.882811 (PMC9533719; doi:10.3389/fpubh.2022.882811)
Supplement: Supplementary file 1 [file Data_Sheet_1.pdf]

Supplementary materials

There are 4 tables and 5 Figures included in this Supplementary materials.

eTable S1. The descriptive statistics about the subjects.
eTable S2. The value range of 48 common measurement features.
eTable S3. Summary of information and methods in the literature related to balance.
eTable S4. The coefficient of determination (R^2) of different methods in different feature dimensions.
eFigure S1. Overall process of balance prediction.
eFigure S2. The Posture diagram of four sets of actions.
eFigure S3. The data set of 61-dimensional features.
eFigure S4. The structure of the proposed neural network model for classification.
eFigure S5. The true and predicted balance score of 100 samples on the test set using the selected 13-dimentional features.

eTable S1. The descriptive statistics about the subjects.

Group (proportion)	Age (mean±std)	Height (mean±std)	Weight (proportion)			
			Underweight	Normal	Overweight	Obese
All	48.86±14.62	164.29±8.40	4.41%	54.63%	33.99%	6.96%
Male (53.64%)	49.13±14.75	169.75±6.23	2.59%	45.08%	43.15%	9.18%
Female(46.36%)	48.56±14.47	157.97±5.73	6.53%	65.69%	23.40%	4.39%
12-20 (0.38%)	18.52±1.94	167.91±7.66	13.89%	69.44%	11.11%	5.56%
21-40 (31.45%)	32.03±4.96	166.85±8.46	7.80%	58.59%	27.11%	6.49%
41-60 (43.99%)	50.47±5.68	163.95±7.87	2.37%	53.44%	37.54%	6.65%
61-80 (24.18%)	68.30±5.26	161.24±8.07	3.06%	50.95%	37.84%	8.15%
	Blood Pressure (proportion)			Balance Ability (proportion)		
	Low	Normal	High	Low	Medium	High
All	6.17%	72.54%	21.29%	20.72%	40.40%	38.88%
Male (53.64%)	2.16%	72.77%	25.07%	25.97%	41.89%	32.14%
Female(46.36%)	10.81%	72.26%	16.93%	14.84%	38.74%	46.42%
12-20 (0.38%)	21.05%	73.68%	5.26%	1.49%	14.93%	83.58%
21-40 (31.45%)	9.56%	83.85%	6.59%	7.43%	34.42%	58.15%
41-60 (43.99%)	5.48%	73.96%	20.55%	19.04%	44.19%	36.76%
61-80 (24.18%)	2.41%	54.25%	43.34%	41.33%	41.69%	16.98%

Notes: among all subjects, the middle-aged and elderly (aged between 41 and 80) accounted for 68.17%. Further, among the people with low and medium balance ability, the middle-aged and elderly (aged between 41 and 80) accounted for 78.36%, indicating that the target population of our proposed method is more suitable for the middle-aged and elderly.

eTable S2. The value range of 48 common measurement features.

Items (units)	FEO	FEC	OFEO	OFEC
Peripheral area (mm^2)	6.6~4203.3	12.0~8515.2	9.6~45410.9	5.2~57102.7
Track length per unit area (mm^2)	0.1~47.6	0.1~27.2	0.0~11.0	0.0~6.2
Total trajectory length of shaking (mm)	18.3~1383.6	43.1~2065.2	83.6~2571.0	26.6~3799.1
Length of track in the X direction (mm)	17.0~1177.9	16.6~1976.5	18.0~1601.4	7.1~2593.1
Length of track in the Y direction (mm)	5.7~975.4	36.7~1800.8	59.1~1961.5	21.3~2407.8
X-axis mean center displacement (mm)	-45.3~67.5	-42.5~65.8	-84.2~100.6	-82.3~114.4
Y-axis mean center displacement (mm)	-68.8~113.4	-68.5~84.1	-119.3~83.0	-120.4~127.1
Maximum dynamic path in the X direction (mm)	1.9~158.8	2.3~207.1	2.0~314.8	1.6~443.4
Maximum dynamic path in the Y direction (mm)	3.7~83.3	5.9~104.6	7.7~231.6	3.4~312.2
Average speed in the X direction (mm/s)	0.6~39.3	0.6~65.9	0.9~80.1	0.7~259.3
Average speed in the Y direction (mm/s)	0.2~32.5	1.2~60.0	3.0~98.1	2.1~240.8
Average yaw speed (mm/s)	0.6~44.7	1.3~67.4	4.0~116.9	2.5~339.5

Notes: The GBSB system uses three-dimensional force measurement to eliminate the interference factor of the individual's weight on the force plate, which can obtain more accurate measurement data. There are four actions measured by the GBSB system, namely 1) stand on feet with eyes open (FEO); 2) stand on feet with eyes closed (FEC); 3) stand on one foot with eyes open (OFEO); 4) stand on one foot with eyes closed (OFEC). Each set of actions includes twelve test items.

eTable S3. Summary of information and methods in the literature related to balance

Ref.	Database	Method	Describe	Performance
Yeh et al. [21]	private	SVM	Balance index: Statokinesigram	Acc: 80.4%
Khandoker et al.[22]	public MFC	SVM	hill-climbing algorithm for feature selection	Acc:78.3%
Begg et al.[23]	public MFC	Gait analysis + SVM	hill-climbing feature selection	Acc:83.3% AUC:77.7%
Scudamore et al.[24]	private	Lasso Regression	functional movement screen score	N/A
Chang et al.[25]	private	SVM	threshold-based classifier	Acc:98.4% Recall:75.6%
Bao et al.[26]	private	SVM	61-dimensional feature	Acc:82.0%
Ahmed et al.[27]	private	CNN+LSTM	using multi-task learning	AUC:75.0%

Notes: MFC: minimum foot clearance dataset; AUC: area under the ROC curve.

eTable S4. The coefficient of determination (R^2) of different methods in different feature dimensions

Method	#F=61	#F=38	#F=21	#F=16	#F=13	#F=12	#F=8
Decision Tree	90.8	85.9	80.6	71.3	69.8	64.9	64.0
Random Forest	95.5	94.3	92.0	91.1	89.4	87.7	84.5
K-Neighbors	86.7	76.5	86.3	84.4	87.8	79.2	79.3
Linear Regression	94.2	93.0	82.8	87.5	87.4	86.7	84.5
Extra Tree	88.7	84.6	84.4	68.2	75.4	70.2	68.0
SVM	90.4	87.5	93.3	81.4	82.7	82.9	78.1
Ours	**97.8**	**96.6**	**95.6**	**92.8**	**92.2**	**90.2**	**88.5**

Notes: #F: the number of feature dimensions. The higher the R^2, the better the model effect. (%). Compared with other methods, our proposed neural network model performs best regardless of the input feature dimension.

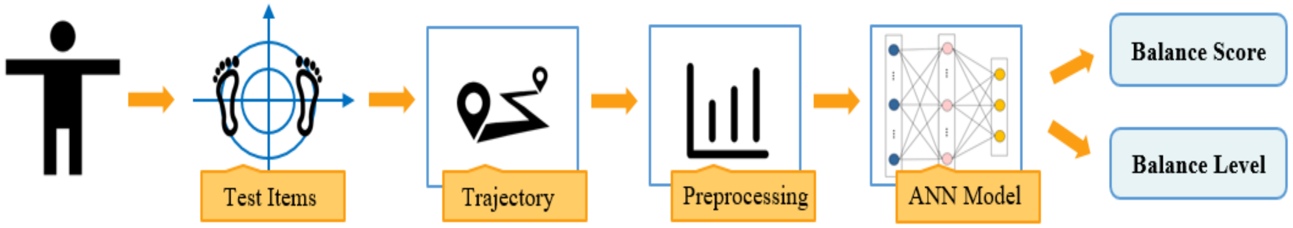

eFigure S1. Overall process of balance prediction.

Notes: subjects need to stand in different positions on the GBSB platform for data collection, Then the system will automatically measure the four sets of items. A lightweight artificial neural network model was proposed to accurately predict the balance ability of new test examples.

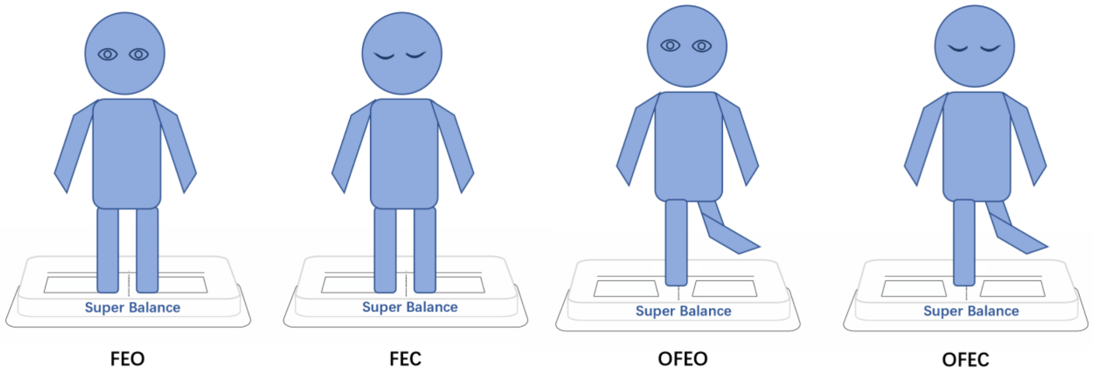

eFigure S2. The Posture diagram of four sets of actions.

Notes: 1) stand on feet with eyes open (FEO); 2) stand on feet with eyes closed (FEC); 3) stand on one foot with eyes open (OFEO); 4) stand on one foot with eyes closed (OFEC).

4 × Common Measurement Features of One Actions + Other Features = The Whole Features

Common Measurement Features of One Actions

1. Peripheral area
2. The average yew speed
3. Track length per unit area
4. Total trajectory length of shaking
5. The average speed in the X direction
6. The average speed in the Y direction
7. The length of track in the X direction
8. The length of track in the Y direction
9. Maximum dynamic path in the X direction
10. Maximum dynamic path in the Y direction
11. X-axis mean center displacement
12. Y-axis mean center displacement

Other Features

1. Age
2. Sex
3. Blood pressure
4. Height
5. Weight
6. Bipedal Romberg
7. Proprioception coefficient
8. Balance stability coefficient
9. Visual adjustment coefficient
10. Proprioception coefficient evaluation
11. Balance stability coefficient evaluation
12. Center of gravity offset
13. Center of gravity offset evaluation

eFigure S3. The data set of 61-dimensional features.

Notes: There are four sets of movements, each of which includes 12-dimensional features, all measured on the GBSB instrument. In addition, there are 13-dimensional unique features, namely age, gender, blood pressure, height, weight, Bipedal Romberg, proprioception coefficient and evaluation, balance stability coefficient and evaluation, visual adjustment coefficient, center of gravity offset and evaluation.

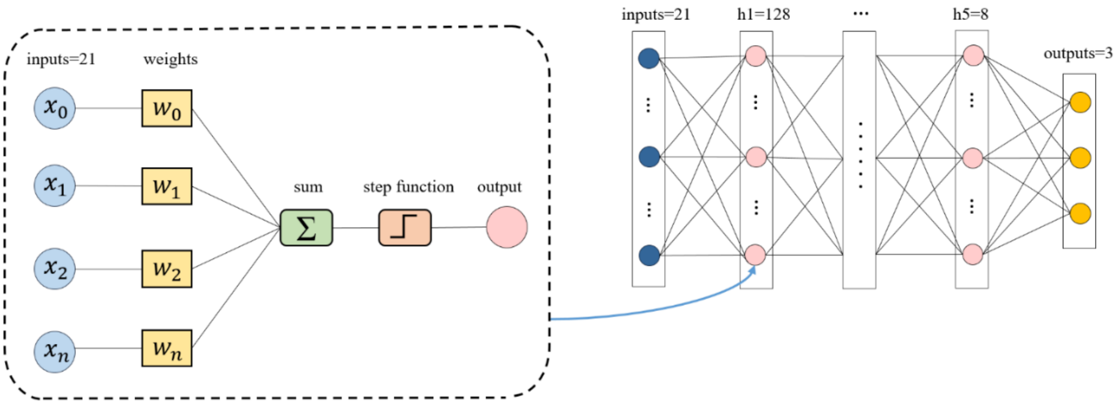

eFigure S4. The structure of the proposed neural network model for classification.

Notes: The entire network contains an input layer, five hidden layers, and an output layer. In each middle-hidden layer, the number of neurons is set as 128, 64, 32, 16, and 8 respectively. The dimension of the input layer matches the number of features of the data, while the output layer represents the prediction result of the neural network.

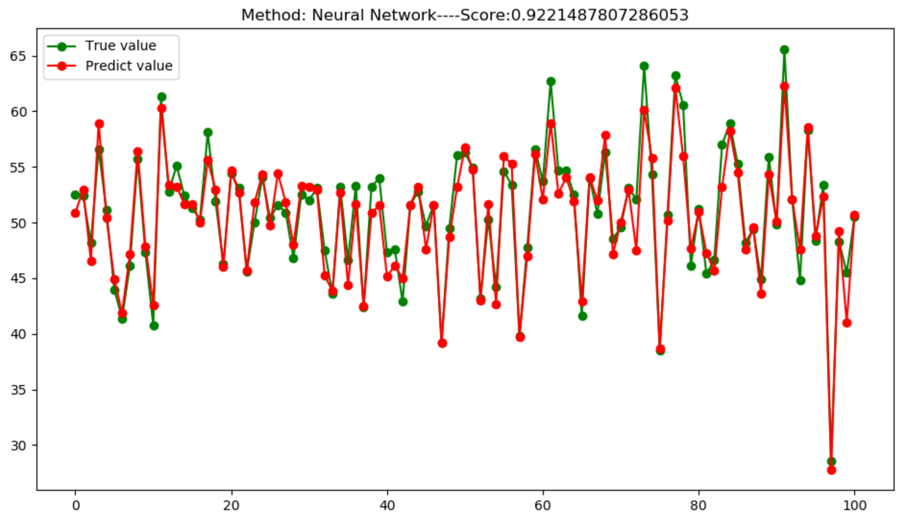

eFigure S5. The true and predicted balance score of 100 samples on the test set using the selected 13-dimentional features.

Notes: In order to show more intuitively, we only drew 100 samples. We can see from the resulting graph that the predicted balance score is basically the same as the true value, which shows the accuracy of the model from an intuitive effect.
